# Effect of non-clinical inter-hospital critical care unit to unit transfer of critically ill patients: a propensity-matched cohort analysis

**DOI:** 10.1186/cc11662

**Published:** 2012-10-03

**Authors:** Helen Barratt, David A Harrison, Kathryn M Rowan, Rosalind Raine

**Affiliations:** 1 Department of Applied Health Research, University College London, 1-19 Torrington Place, London WC1E 6BT, UK; 2 Intensive Care National Audit & Research Centre, Napier House, 24 High Holborn, London WC1V 6AZ, UK

## Abstract

**Introduction:**

No matter how well resourced, individual hospitals cannot expect to meet all peaks in demand for adult general critical care. However, previous analyses suggest that patients transferred for non-clinical reasons have worse outcomes than those who are not transferred, but these studies were underpowered and hampered by residual case-mix differences. The aim of this study was to evaluate the effect of transferring adult general critical care patients to other hospitals for non-clinical reasons.

**Methods:**

We carried out a propensity-matched cohort analysis comparing critical care patients who underwent a non-clinical critical care unit to unit transfer to another hospital with those who were not transferred. The primary outcome measure was mortality at ultimate discharge from acute hospital. Secondary outcomes were mortality at ultimate discharge from critical care, plus length of stay in both critical care and acute hospital.

**Results:**

A total of 308,323 patients were admitted to one of 198 adult general critical care units in England and Wales between January 2008 and September 2011. This included 759 patients who underwent a non-clinical transfer within 48 hours of admission to the unit and 1,518 propensity-matched patients who were not transferred. The relative risk of ultimate acute hospital mortality was 1.01 (95% confidence interval = 0.87 to 1.16) for the non-clinical transfer group, compared with patients who were not transferred but had a similar propensity for transfer. There was no statistically significant difference in ultimate critical care unit mortality. Transferred patients received on average three additional days of critical care (*P *< 0.001) but the difference in length of acute hospital stay was of only borderline significance (*P *= 0.05).

**Conclusion:**

In our analysis the difference in mortality between non-clinical transferred and nontransferred patients was not statistically significant. Nevertheless, non-clinical transfers received, on average, an additional 3 days of critical care. This has potential ramifications in terms of distress, inconvenience and cost for patients, their families, and the National Health Service. We therefore need further evidence, including qualitative data from family members and cost-effective analyses, to better understand the broader effects of non-clinical transfer.

## Introduction

When there are no critical care beds available locally, patients may require transfer to another hospital to receive care. Adverse incidents may occur in up to one-third of inter-hospital transfers, however, and so measures have been established to contain non-clinical transfers in local networks of critical care units [1-3]. The most recent patient transfer guidance issued in the UK recommends that transfers for capacity reasons should occur only as a last resort [4]. Uncertainty remains, however, about the impact of non-clinical transfers on patients. Previous analyses have suggested that transferred patients have higher mortality rates and require longer hospital stays [5,6]. However, these studies include patients transferred to access regional specialist services (for example, neurosurgery); such transfers will always occur with regionalisation of these services. Patients in this group are likely to be more seriously ill than the average critical care patient and thus their outcomes may not be applicable to those transferred for non-clinical reasons.

The only published study of non-clinical transfers examined patients admitted to hospitals in the state of Victoria, Australia. The 75 transferred patients had significantly longer lengths of stay in critical care and hospital. The analysis was underpowered to detect a difference in hospital mortality and the study was limited by difficulties in finding matched control patients [7]. With a relatively small pool of potential control patients, it is usually only practical to match on a limited number of covariates. Even if matching is successful, there may still be large underlying differences between the two groups, confounding the estimate of effect [8]. Randomisation would raise ethical issues, but propensity-based methods offer a way forward. They provide a better balance of covariates and have been used with success on critical care data [9-11]. Patient characteristics are used to derive the predicted probability - or propensity - of a patient receiving an intervention, regardless of whether they received the intervention or not. The propensity is calculated as a single figure for each patient using regression methods, which can accommodate a large number of potential confounders. Cases that received the intervention are matched with controls that did not receive the intervention but had a similar propensity. Although individual matched groups of cases and controls may differ in terms of specific characteristics (for example, gender), this approach aims to provide balance on patient characteristics across the study population.

We used propensity-based methods to evaluate the effect of non-clinical, critical care unit to unit transfer at the individual patient level. We compared mortality and length of stay in critical care patients who underwent a non-clinical transfer within 48 hours of admission to the original critical care unit with a cohort of patients admitted during the same period who were not transferred.

## Materials and methods

### Case Mix Programme

The Case Mix Programme (CMP) is the national clinical audit of adult, general critical care units (including intensive care and combined intensive care and high-dependency units) in England, Wales and Northern Ireland. The CMP is coordinated by the Intensive Care National Audit & Research Centre (ICNARC). Coverage of the database increased from over 60% of eligible units in 2008 to over 80% in 2010. Raw clinical data are abstracted retrospectively by trained local data collectors in accordance with precise rules and definitions. The data then undergo extensive validation, both locally and centrally. The CMP data collection and validation processes have been previously reported [12] and independently assessed to be of high quality [13]. CMP data collection covers the first 24-hour case mix (demographics, past medical history, surgical status, acute severity of illness and reason for admission) and outcomes (critical care unit and acute hospital discharge status). Support for the collection and use of patient-identifiable data without consent was obtained under Section 251 of the UK NHS Act 2006 (approval number: PIAG 2-10[f]/2005).

Patients aged 16 years and older were eligible for inclusion if they were admitted to an adult, general critical care unit in England or Wales that was participating in the CMP between 1 January 2008 and 30 September 2011. Subsequent critical care admissions to the same unit within the same hospital stay were excluded. Patients admitted to critical care units in Northern Ireland were also excluded because a comparable measure of socioeconomic position was not available.

### Selection of cases and controls

Inter-hospital transfers were identified as patients discharged from the critical care unit to a level 3 bed in another acute hospital. Level 3 care is defined as the level of care for 'patients requiring advanced respiratory support alone or basic respiratory support together with support of at least two organ systems'. This level includes all complex patients requiring support for multiorgan failure [3]. Non-clinical transfers were defined as transfers for whom the reason for discharge from the original critical care unit was reported as transferred for comparable critical care (that is, for similar care as provided in the transferring unit). The CMP does not capture any additional information about the reason for transfer.

As acute severity of illness data are recorded in the first 24 hours following admission to a critical care unit in the CMP, patients transferred within 48 hours were selected to minimise the likelihood of acute severity of illness changing markedly in the time since assessment. The analysis was therefore restricted to patients who underwent a non-clinical transfer within 48 hours of admission to the original critical care unit.

A propensity model was built using logistic regression to model factors predictive of undergoing a non-clinical transfer, including patient and unit factors thought to be relevant on the basis of previous analyses. Patient factors included were: age; sex; deprivation; past medical history; admission type; surgical status; primary reason for admission to the critical care unit; acute severity of illness; month of admission to the critical care unit; and time of day of admission to the critical care unit.

Deprivation was assessed using area-based measures: the English Index of Multiple Deprivation (IMD) 2010 [14] and the Welsh IMD 2008 [15]. Each lower layer super output area (about 1,500 people) was assigned a deprivation score. Scores were then ranked and divided into quintiles (1 for least deprived, 5 for most deprived) and each patient was assigned to an IMD quintile based on their postcode of residence.

Past medical history was assessed against a list of specified, serious conditions, evident during the 6 months prior to admission to the critical care unit, and defined by the Acute Physiology and Chronic Health Evaluation II method [16]. Admission type was coded as per the Critical Care Minimum Data Set [17]. Surgical admissions were identified by admission to the critical care unit direct from theatre and recovery, and were subclassified as either elective/scheduled or emergency/urgent based on the National Confidential Enquiry into Perioperative Deaths classification [18].

The primary reason for admission to the critical care unit was coded using the ICNARC Coding Method [19] and was categorised by the underlying body system involved (for example, respiratory, neurological, and so forth). Acute severity of illness was assessed using the ICNARC Physiology Score, based on 12 physiological parameters measured during the first 24 hours in the critical care unit [20]. Unit factors included were the number of critical care beds in the transferring unit, and the critical care network of the transferring unit. Components of each of the variables are outlined in Table 1.

**Table 1:** Adjusted odds ratios for patients undergoing non-clinical transfer within 48 hours of critical care admission

	Adjusted odds ratio	95% confidence interval	*P *value
Sex			
Male versus female	1.00	0.87 to 1.16	0.95
Age	See Figure 1	< 0.001	
Quintiles of IMD			
1 (least deprived)	1	-	0.95
2	0.97	0.75 to 1.26	
3	0.99	0.77 to 1.28	
4	1.06	0.83 to 1.35	
5 (most deprived)	0.98	0.77 to 1.25	
Past medical history of one or more specified serious conditions^a^			
Yes versus no	0.96	0.79 to 1.17	0.70
Admission type			
Unplanned local admission	1	-	< 0.001
Planned local medical admission	0.89	0.42 to 1.90	
Planned local surgical admission	0.33	0.16 to 0.68	
Unplanned transfer in	1.16	0.82 to 1.64	
Planned transfer in	1.84	1.31 to 2.59	
Repatriation	0.36	0.13 to 0.96	
Surgical status			
Nonsurgical	1	-	< 0.001
Emergency/urgent	0.49	0.38 to 0.64	
Elective/scheduled	0.23	0.12 to 0.42	
Reason for admission by body system			
Cardiovascular	1	-	< 0.001
Respiratory	1.00	0.80 to 1.25	
Neurological	1.51	1.17 to 1.94	
Gastrointestinal	0.81	0.61 to 1.06	
Genitourinary including renal	0.63	0.45 to 0.87	
Haematological/immunological	0.68	0.36 to 1.31	
Endocrine, metabolic, thermoregulation and poisoning	0.66	0.46 to 0.91	
Musculoskeletal	0.98	0.59 to 1.65	
Dermatological	1.12	0.57 to 2.22	
ICNARC Physiology Score	See Figure 2	< 0.001	
Month of admission to critical care			
January	1	-	0.003
February	0.70	0.51 to 0.95	
March	0.80	0.59 to 1.06	
April	0.71	0.53 to 0.97	
May	0.68	0.50 to 0.92	
June	0.55	0.40 to 0.77	
July	0.50	0.35 to 0.72	
August	0.73	0.53 to 1.00	
September	0.54	0.38 to 0.77	
October	0.61	0.44 to 0.86	
November	0.61	0.43 to 0.85	
December	0.80	0.59 to 1.08	
Time of admission to critical care			
07:00 to 18:59	1	-	0.24
19:00 to 23:59	0.86	0.72 to 1.03	
00:00 to 06:59	0.93	0.78 to 1.11	
Number of beds in referring critical care unit	0.96	0.94 to 0.98	< 0.001

ICNARC, Intensive Care National Audit and Research Centre; IMD, Index of Multiple Deprivation. ^a^The conditions included in the variable 'past medical history of one or more specified serious conditions' are: biopsy-proven cirrhosis, portal hypertension, hepatic encephalopathy, very severe cardiovascular disease (New York Heart Association functional class IV), severe respiratory disease (permanent shortness of breath with light activity), home ventilation, chronic renal replacement therapy, AIDS, daily high-dose steroid treatment, radiotherapy, chemotherapy, metastatic disease, acute/chronic myelogenous/lymphocytic leukaemia or multiple myeloma, lymphoma, or congenital immunohumoral or cellular immune deficiency state.

Continuous variables (age and ICNARC Physiology Score) were modelled using restricted cubic splines to allow for a flexible, nonlinear relationship between variables [21]. The model was then used to calculate a propensity of 0 to 1 for each eligible patient. Each patient undergoing a non-clinical transfer (case) was matched on the basis of absolute propensity (nearest-neighbour match) with two patients who were not transferred, but who had a length of stay at least as long as the time point at which the non-clinical transfer took place (controls). Sampling was carried out with replacement; patients thus may have acted as a control for more than one case.

### Analysis

To ensure that there was similarity between the two groups on important case-mix factors, the balance of patient-level factors between cases and matched controls was assessed using the chi-square test for categorical variables and using both quantile-quantile plots and the Kolmogorov-Smirnov test for continuous variables. The mean square difference in propensity between cases and controls was also calculated to assess the closeness of matching.

Comparisons of mortality were performed using conditional fixed-effects Poisson regression with 95% confidence intervals (CIs) estimated by bootstrap resampling with 500 replications (effect estimate: matched groups relative risk) [22]. Regression models were adjusted for the ICNARC model predicted log-odds of hospital mortality to further account for any residual differences in acute severity of illness at admission. In order to assess whether there was a significant change in acute severity of illness in the time between admission and transfer, we measured the difference in ICNARC Physiology Score in those cases in which it was possible to link data from their initial admission to critical care to information from their subsequent admission to a second unit following transfer. Comparisons of total acute hospital length of stay and total critical care unit length of stay were performed using repeated-measures analysis of variance.

The primary outcome was mortality at ultimate discharge from acute hospital (ultimate acute hospital mortality). Secondary outcomes were mortality at ultimate discharge from critical care (ultimate critical care unit mortality), total acute hospital length of stay and total critical care unit length of stay.

Statistical analyses were performed using Stata 10.1 (StataCorp LP, College Station, TX, USA).

## Results

### Study population

During the study period, 308,323 eligible patients, aged 16 or over, were admitted to 198 participating adult general critical care units in England and Wales. These patients were admitted to critical care for the first time during that hospital stay. IMD quintiles could not be assigned to 2,578 (0.8%) patients and a further 11 (0.004%) were excluded because they were missing information about their source of admission. An additional 3,102 patients (1.0%) were excluded because they had missing information relating to one or more of the key outcome variables, whilst 1,577 patients (0.5%) were excluded because they had undergone a non-clinical transfer more than 48 hours after admission to critical care. We have provided additional data about the distribution of time from critical care unit admission to non-clinical transfer in the transferred population in Figure S1 of Additional File 1. Table S1 in Additional File 1 also presents information about the characteristics of patients undergoing a non-clinical transfer within 48 hours of admission to critical care, compared with after 48 hours. A propensity was calculated for 301,055 patients (97.6%), including 759 patients transferred for non-clinical reasons less than 48 hours after admission.

**Figure 1:**
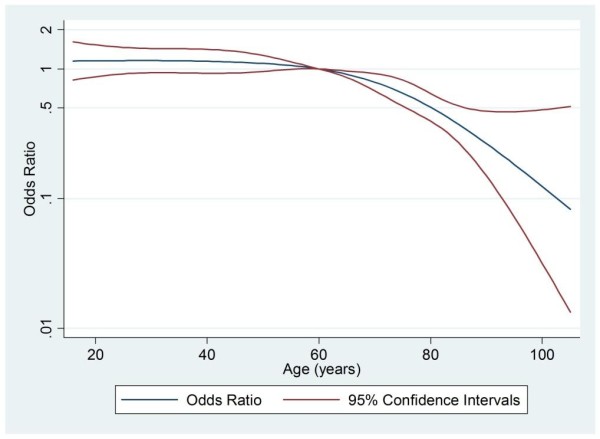
**Adjusted odds ratio for non-clinical transfer by age**. Adjusted odds ratio for patients undergoing non-clinical transfer within 48 hours of admission to critical care by age relative to age 60 years.

**Figure 2:**
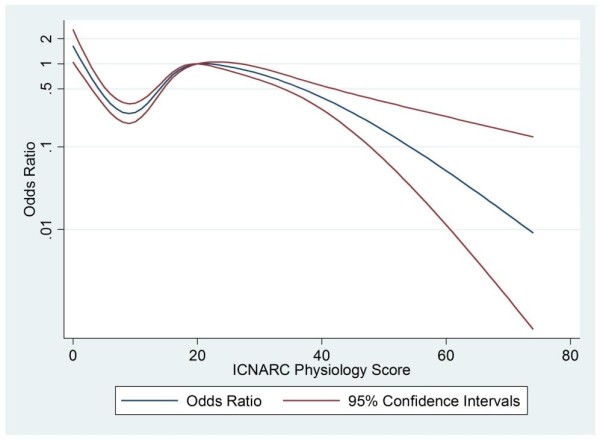
**Adjusted odds ratio for non-clinical transfer by physiology score**. Adjusted odds ratio for patients undergoing non-clinical transfer within 48 hours of admission to critical care by Intensive Care National Audit and Research Centre (ICNARC) Physiology Score relative to a score of 20.

Table 1 and Figures 1 and 2 together show the key patient and unit-level factors that were predictive of a patient undergoing a non-clinical transfer, as demonstrated by the logistic regression analysis used to build the propensity model. Figures 1 and 2 illustrate the odds ratio of undergoing transfer by both age and physiology score relative to the mean of each variable as a baseline. The likelihood of transfer varies significantly with both age and physiology score. When the analysis was adjusted for all other factors, surgical patients, both emergency and elective, were significantly less likely to undergo a non-clinical transfer, as were those with endocrine or genitourinary (including renal) causes for admission. Patients admitted for neurological reasons were more likely to undergo a non-clinical transfer. In addition, the odds of patients undergoing a non-clinical transfer varied significantly throughout the year, being highest in January and lowest in July. In terms of unit-level factors, the odds of transfer decreased as the number of critical care beds in the transferring unit increased.

The odds of patients undergoing a non-clinical transfer were also calculated for each of 28 critical care networks in England and Wales. Compared with a randomly chosen baseline network, the adjusted odds ratio for non-clinical transfer within 48 hours varied from 0.29 (95% CI = 0.13 to 0.68) to 4.90 (95% CI = 3.04 to 7.90; *P *< 0.001).

### Matching on propensity

**Figure 3:**
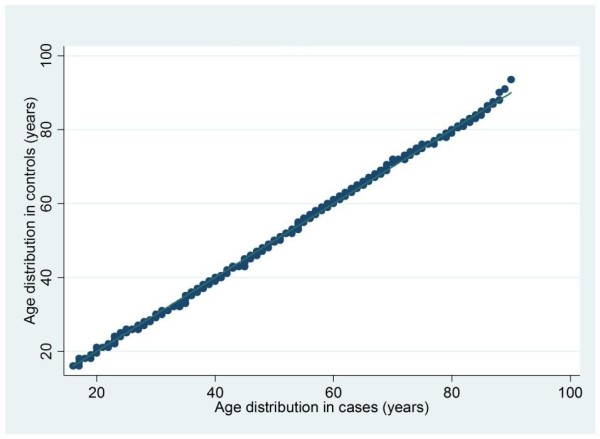
**Quantile-quantile plot comparing the distribution of age in cases and controls**. Quantile-quantile plot comparing the distribution of age in patients undergoing a non-clinical transfer within 48 hours of admission to critical care (cases) and matched patients not undergoing transfer (controls).

**Figure 4:**
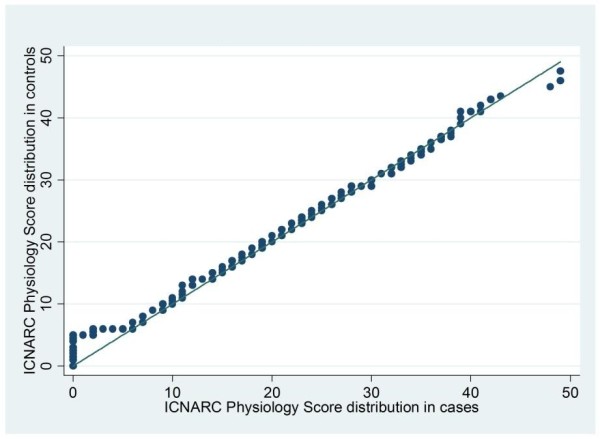
**Quantile-quantile plot comparing the distribution of physiology scores in cases and controls**. Quantile-quantile plot comparing Intensive Care National Audit and Research Centre Physiology Scores in cases and controls.

Each case was successfully matched with two controls. There was good balance between the two groups (Table 2 and Figures 3 and 4), with no significant differences in distributions. Only 11 controls (0.7%) were matched to two different cases and none were matched to more than two cases. The mean square difference in propensity between cases and controls was < 0.00001, giving assurance that the propensity matches were very close.

**Table 2:** Characteristics of patients undergoing non-clinical transfer compared with matched patients not undergoing transfer

	Non-clinical transfer patients (*n *= 759)	Matched patients not undergoing transfer (*n *= 1,518)	*P *value^a^
Age (years)	55.7 (17.5)	55.7 (17.7)	0.99
Sex			
Female	328 (43.2%)	660 (43.5%)	0.91
Male	431 (56.8%)	858 (56.5%)	
Quintiles of IMD			
1 (least deprived)	107 (14.1%)	231 (15.2%)	0.82
2	121 (15.9%)	241 (15.9%)	
3	140 (18.5%)	297 (19.6%)	
4	179 (23.6%)	330 (21.7%)	
5 (most deprived)	212 (27.9%)	419 (27.6%)	
Past medical history of one or more specified serious conditions			
No	629 (82.9%)	1,278 (84.2%)	0.42
Yes	130 (17.1%)	240 (15.8%)	
Admission type			
Unplanned local admission	664 (87.5%)	1,318 (86.8%)	0.75
Planned local medical admission	7 (0.9%)	10 (0.7%)	
Planned local surgical admission	12 (1.6%)	19 (1.3%)	
Unplanned transfer in	35 (4.6%)	90 (5.9%)	
Planned transfer in	37 (4.9%)	71 (4.7%)	
Repatriation	4 (0.5%)	10 (0.7%)	
Surgical status			
Nonsurgical	669 (88.1%)	1,315 (88.6%)	0.37
Emergency/urgent	73 (9.6%)	174 (11.5%)	
Elective/scheduled	17 (2.2%)	29 (1.9%)	
Reason for admission by body system			
Cardiovascular	119 (15.7%)	247 (16.3%)	0.97
Respiratory	248 (32.7%)	508 (33.5%)	
Neurological	152 (20.0%)	286 (18.8%)	
Gastrointestinal	103 (13.6%)	197 (13.0%)	
Genitourinary including renal	50 (6.6%)	98 (6.5%)	
Haematological/immunological	10 (1.3%)	25 (1.7%)	
Endocrine, metabolic, thermoregulation and poisoning	51 (6.7%)	108 (7.1%)	
Musculoskeletal	17 (2.2%)	26 (1.7%)	
Dermatological	9 (1.2%)	23 (1.5%)	
ICNARC Physiology Score	20.1 (8.3)	20.6 (7.8)	0.31

Characteristics of patients undergoing non-clinical transfer within 48 hours of admission to critical care compared with matched patients not undergoing transfer. Data presented as *n *(%) or mean (standard deviation). ICNARC, Intensive Care National Audit and Research Centre; IMD, Index of Multiple Deprivation. ^a^Chi-square test for categorical variables; Kolmogorov-Smirnov test for continuous variables.

To measure the change in acute severity of illness between the initial admission to critical care and following the transfer, we were able to link CMP data for 460/759 cases (60.6%). The mean ICNARC Physiology Score at initial admission to critical care was 20.6; the mean score on admission to the second unit after non-clinical transfer was 19.8 (*P *= 0.06).

### Mortality

The crude acute hospital and critical care unit mortality were both slightly lower in the non-clinical transfer patients, compared with the propensity-matched controls (Table 3). In the propensity-matched analysis, adjusted for the ICNARC model predicted log-odds of hospital mortality, the relative risk of ultimate acute hospital mortality was 1.01 (95% CI = 0.87 to 1.16; *P *= 0.94) in patients who had undergone a non-clinical transfer within 48 hours following admission, compared with those who were not transferred. The relative risk of ultimate critical care unit mortality for nontransferred patients was 0.98 (95% CI = 0.82 to 1.18; *P *= 0.84).

**Table 3:** Crude outcomes among patients undergoing non-clinical transfer compared with propensity-matched patients not undergoing transfer

	Non-clinical transfer patients (*n *= 759)	Propensity-matched patients not undergoing transfer (*n *= 1,518)
Ultimate acute hospital mortality	223 (29.4%)	471 (31.0%)
Ultimate critical care unit mortality	162 (21.3%)	345 (22.7%)
Total critical care unit length of stay (days)	11.0 (16.9)/6 (2 to 14)	7.8 (9.7)/4 (2 to 9)
Acute hospital length of stay (days)	29.8 (36.4)/19 (9 to 37)	26.9 (33.8)/16 (7 to 33)

Crude outcomes among patients undergoing a non-clinical transfer within 48 hours of admission to critical care compared with propensity matched patients not undergoing transfer. Data presented as *n *(%) or mean (standard deviation)/median (interquartile range).

### Length of stay

The mean total length of stay in critical care was 3.2 days longer (95% CI = 2.1 to 4.3; *P *< 0.001) in the non-clinical transfer group. The mean total acute hospital length of stay was 3.0 days longer (95% CI = -0.1 to 6.1; *P *= 0.06).

## Discussion

In our analysis, there was no statistically significant difference in either ultimate acute hospital mortality or ultimate critical care mortality for patients transferred to another acute hospital for comparable critical care, within 48 hours of admission to the original critical care unit, compared with those who were not. However, the 95% CI included a 13% lower risk to a 16% higher risk, and therefore we cannot exclude a potential level of harm that could be considered clinically significant. In addition, patients transferred for non-clinical reasons received, on average, an additional 3 days of critical care compared with those who were not.

Alongside the risk of adverse incidents outside the critical care environment, moving patients can generate additional physiological stress during transfer [23]. These changes are transient, however, and are not captured by our analysis. The additional 3 days of critical care required by the transfer group may be because patients have to regain their physiological stability following transfer.

In 2000, measures were established to contain non-clinical transfers in local networks of critical care units [3]. Our results demonstrated a 16-fold variation in the likelihood of patients undergoing a non-clinical transfer within 48 hours across the 28 critical care networks in England and Wales (range of adjusted odds ratios 0.29 to 4.90) in the period 1 January 2008 to 30 September 2011. Our findings also showed that the likelihood of patients undergoing a transfer varied with the number of critical care beds in the referring unit. However, the variation between networks maybe the result of differences in either critical care provision or local policies aimed at reducing non-clinical transfers

### Comparison with other studies

Our analysis has several strengths when compared with the previous study [7]. First, we examined data on a large number of patients transferred for comparable critical care excluding those transferred for more specialised critical care. Second, given the high level of critical care unit participation, the CMP data are highly representative of patient experience in England and Wales. Third, the use of a propensity-based method enabled us to address the challenge of potential confounding that has hampered previous analyses. Fourth, we were able to closely match all 759 patients transferred for non-clinical reasons with two control patients sharing a similar propensity to undergo a non-clinical transfer. This approach enabled us to incorporate a greater number of possible confounders in the matching process than is feasible when using conventional methods.

### Limitations of the study

Our analysis only included patients transferred between critical care units. However, we know that some patients are transferred directly from the emergency department of one hospital to a critical care unit in another hospital [24]. These patients are not captured by the CMP, but this group is likely to be sicker and less stable clinically. Further, quantifying the impact of non-clinical transfer in all patients, including those transferred from the emergency department, would require a large, prospective study in which acute severity of illness was recorded regularly so that cases could be matched based on the severity of their condition at the time of transfer. Given the relatively small number of non-clinical transfers that take place, this would need to be coordinated across a large number of centres, over many months, to ensure sufficient numbers of patients, making the study challenging and potentially unfeasible.

The CMP exists for national clinical audit purposes and only requires physiological data to calculate acute severity of illness scores in the 24 hours following admission to the critical care unit. We therefore restricted our analysis solely to those patients transferred for non-clinical reasons closer to this time period. However, omitting an unmeasured, yet potentially confounding, variable from the propensity model has been shown to result in biased estimation of the treatment effect [11]. To this end, there is a risk of residual confounding in our analysis associated with the timing of the measurement of acute severity of illness. Patients selected for non-clinical transfer may have a lower acute severity of illness at the time of transfer, compared with other patients in the critical care unit, and thus a lower risk of mortality. However, by restricting our analysis to non-clinical transfers within 48 hours of admission, there is less opportunity for the severity of illness to change markedly from the first 24 hours, so the risk of bias is minimised. In the group of transferred patients whose data could be linked between two critical care units, there was no statistically significant difference in acute severity of illness in the 24 hours following the second admission after transfer compared with the same period after initial admission. Another potential limitation is that patients transferred within 48 hours constitute only about one-third of all the non-clinical transfers that occurred and may therefore not be representative of all non-clinical transfers. Additionally, the difference in total critical care length of stay may be partly attributable to a selection bias; for example, if clinicians select a patient for non-clinical transfer who they consider is likely to require several more days of critical care.

## Conclusion

Organisations including the UK Intensive Care Society have recommended that transfers for capacity reasons should only occur as a last resort, in part because of evidence about the risk of adverse events and the difficulties of delivering care outside the critical care setting [4]. However, in our analysis of those patients transferred within 48 hours of admission to critical care, we demonstrate no statistically significant difference in either ultimate acute hospital mortality or ultimate critical care mortality between transferred and nontransferred patients. Nevertheless, non-clinical transfers received, on average, an additional 3 days of critical care. This has potential ramifications in terms of distress, inconvenience and cost for patients, their families, and the National Health Service. We therefore need further evidence, including qualitative data from family members and cost-effective analyses, to better understand the broader effects of non-clinical transfer.

## Key messages

• No matter how well resourced, individual hospitals cannot expect to meet all peaks in demand for adult general critical care, but transferring patients outside the critical care environment involves risk.

• In our analysis there was no statistically significant difference in ultimate acute hospital mortality in patients transferred for non-clinical reasons, but we cannot rule out a level of harm that may be considered clinically significant.

• Transferred patients also received, on average, three additional days of critical care.

• Non-clinical transfers may also involve an additional burden in terms of distress, inconvenience and cost for patients and their families. We need further evidence - for example, from qualitative interviews with family members - to understand the broader effects of non-clinical transfer.

## Abbreviations

CI: confidence interval; CMP: Case Mix Programme; ICNARC: Intensive Care National Audit and Research Centre; IMD: Index of Multiple Deprivation.

## Competing interests

The authors declare that they have no competing interests.

## Authors' contributions

All authors took part in planning the study. HB and DAH analysed the data. All authors took part in interpreting the results and reporting the research. All authors read and approved the manuscript for publication.

## Supplementary Material

Additional file 1 **Supplementary information relevant to the study**. Table S1 presenting a comparison of characteristics of patients undergoing a non-clinical transfer within 48 hours of admission to critical care with those after 48 hours. Figure S1 showing an assessment of the distribution of time from critical care unit admission to non-clinical transfer in the study population. Click here for file
