# Supplementary material for: Effect of non-clinical inter-hospital critical care unit to unit transfer of critically ill patients: a propensity-matched cohort analysis
Source: Crit Care. 2012 Oct 3;16(5):R179. doi: 10.1186/cc11662 (PMC3682280; doi:10.1186/cc11662)

**Supplementary information**

The paper describes how we measured the difference in ICNARC Physiology Score in those cases in which it was possible to link data from their initial admission to critical care to information from their subsequent admission to a second unit following transfer. For comparison, we have repeated this for those patients who were transferred more than 48 hours after admission to critical care, to examine whether acute severity of illness has changed more in this group. We successfully linked 73% of patients transferred >48h after admission, and the ICNARC Physiology Score was on average 5 points lower on arrival at the second unit in this group (p<0.001). [The change in acute severity of illness in those transferred less than 48 hours after admission to critical care was less than one point (p=0.06).]

**Table S1: Characteristics of patients undergoing a non-clinical transfer within 48 hours of admission to critical care compared with after 48 hours**

|  | **Non-clinical transfer within  48 hours**  **(n= 759)** | **Non-clinical transfer after  48 hours**  **(n=** **1577)** |
| --- | --- | --- |
|  |  |  |
| **Age (years), mean (SD)** | 55.7 (17.5) | 58.9 (16.0) |
|  |  |  |
| **Sex, n (%)** |  |  |
| Female | 328 (43.2) | 646 (41.0) |
| Male | 431 (56.8) | 931 (59.0) |
|  |  |  |
| **Quintiles of IMD, n (%)** |  |  |
| 1 (least deprived) | 107 (14.1) | 219 (13.9) |
| 2 | 121 (15.9) | 242 (15.3) |
| 3 | 140 (18.5) | 277 (17.6) |
| 4 | 179 (23.6) | 362 (23.0) |
| 5 (most deprived) | 212 (27.9) | 477 (30.2) |
|  |  |  |
| **Past medical history of one or more specified serious conditions, n (%)** |  |  |
| No | 629 (82.9) | 1,322 (83.8) |
| Yes | 130 (17.1) | 255 (16.2) |
|  |  |  |
| **Admission type, n (%)** |  |  |
| Unplanned local admission | 664 (87.5) | 1,180 (74.8) |
| Planned local medical admission | 7 (0.9) | 12 (0.8) |
| Planned local surgical admission | 12 (1.6) | 37(2.3) |
| Unplanned transfer in | 35 (4.6) | 173 (11.0) |
| Planned transfer in | 37 (4.9) | 154 (9.8) |
| Repatriation | 4 (0.5) | 21 (1.3) |
|  |  |  |
| **Surgical status, n (%)** |  |  |
| Non-surgical | 669 (88.1) | 1,359 (86.2) |
| Emergency/Urgent | 73 (9.6) | 179 (11.4) |
| Elective/Scheduled | 17 (2.2) | 39 (2.5) |
|  |  |  |
| **Reason for admission by body system, n (%)** |  |  |
| [Cardiovascular](javascript:__doPostBack('ICM1$ICM1','s0\\2')) | 119 (15.7) | 205 (13.0) |
| [Respiratory](javascript:__doPostBack('ICM1$ICM1','s0\\1')) | 248 (32.7) | 698 (44.3) |
| [Neurological](javascript:__doPostBack('ICM1$ICM1','s0\\4')) | 152 (20.0) | 181 (11.5) |
| [Gastrointestinal](javascript:__doPostBack('ICM1$ICM1','s0\\3')) | 103 (13.6) | 270 (17.1) |
| [Genito-urinary](javascript:__doPostBack('ICM1$ICM1','s0\\7')) including renal | 50 (6.6) | 102 (6.5) |
| [Haematological/Immunological](javascript:__doPostBack('ICM1$ICM1','s0\\9')) | 10 (1.3) | 16 (1.0) |
| [Endocrine, Metabolic, Thermoregulation and   Poisoning](javascript:__doPostBack('ICM1$ICM1','s0\\8')) | 51 (6.7) | 46 (2.9) |
| [Musculoskeletal](javascript:__doPostBack('ICM1$ICM1','s0\\10')) | 17 (2.2) | 48 (3.0) |
| [Dermatological](javascript:__doPostBack('ICM1$ICM1','s0\\11')) | 9 (1.2) | 11 (0.7) |
|  |  |  |
| **ICNARC Physiology Score, mean (SD)** | 20.1 (8.3) | 23.3 (7.6) |
|  |  |  |

**Figure S1: Distribution of time from critical care unit admission to non-clinical transfer**


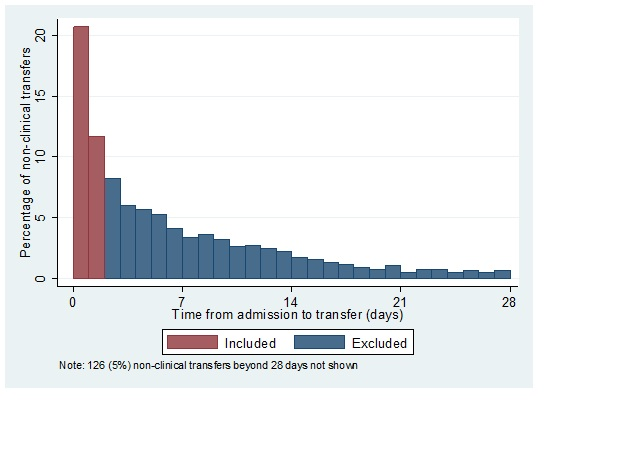

Supplement: Additional file 1 — Supplementary information relevant to the study. Table S1 presenting a comparison of characteristics of patients undergoing a non-clinical transfer within 48 hours of admission to critical care with those after 48 hours. Figure S1 showing an assessment of the distribution of time from critical care unit admission to non-clinical transfer in the study population. [file cc11662-S1.DOCX]
